# Bridgehead Effect in the Worldwide Invasion of the Biocontrol Harlequin Ladybird

**DOI:** 10.1371/journal.pone.0009743

**Published:** 2010-03-17

**Authors:** Eric Lombaert, Thomas Guillemaud, Jean-Marie Cornuet, Thibaut Malausa, Benoît Facon, Arnaud Estoup

**Affiliations:** 1 Equipe “Biologie des Populations en Interaction”, INRA UMR 1301 IBSV (INRA/CNRS/Université de Nice-Sophia Antipolis), Sophia-Antipolis, France; 2 INRA UMR Centre de Biologie et de Gestion des Populations (INRA/IRD/Cirad/Montpellier SupAgro), Montferrier-sur-Lez, France; Centre National de la Recherche Scientifique, France

## Abstract

Recent studies of the routes of worldwide introductions of alien organisms suggest that many widespread invasions could have stemmed not from the native range, but from a particularly successful invasive population, which serves as the source of colonists for remote new territories. We call here this phenomenon the invasive bridgehead effect. Evaluating the likelihood of such a scenario is heuristically challenging. We solved this problem by using approximate Bayesian computation methods to quantitatively compare complex invasion scenarios based on the analysis of population genetics (microsatellite variation) and historical (first observation dates) data. We applied this approach to the Harlequin ladybird *Harmonia axyridis* (HA), a coccinellid native to Asia that was repeatedly introduced as a biocontrol agent without becoming established for decades. We show that the recent burst of worldwide invasions of HA followed a bridgehead scenario, in which an invasive population in eastern North America acted as the source of the colonists that invaded the European, South American and African continents, with some admixture with a biocontrol strain in Europe. This demonstration of a mechanism of invasion via a bridgehead has important implications both for invasion theory (i.e., a single evolutionary shift in the bridgehead population versus multiple changes in case of introduced populations becoming invasive independently) and for ongoing efforts to manage invasions by alien organisms (i.e., heightened vigilance against invasive bridgeheads).

## Introduction

Elucidating the routes and modalities of introductions of undesirable organisms is crucial for managers who wish to prevent new invasions. [1], [2]. It is also a prerequisite to generating and testing useful hypotheses regarding the environmental and evolutionary factors responsible for biological invasions [3]. In the large body of literature on biological invasions, a number of studies suggest that successful invasions involve a particular invasive population, which serves as the source of colonists for remote new territories [4], [5], [6], [7], [8]. We call here this phenomenon the *invasive bridgehead effect*, likening the successful population of a biological invader to a military force that establishes a foothold, historically at the far side of a bridge, prior to further incursions into hostile territories. Convincing demonstrations of such an invasive bridgehead effect are however scarce because of the lack of appropriate methods to confidently reconstruct the routes of invasion and hence formally test this scenario against alternative ones. Moreover, too few invasive populations have been studied to capture the global picture of the worldwide invasion process for most species.

The harlequin ladybird *Harmonia axyridis* (HA), or multicolored Asian lady beetle, is an appropriate biological model to test for the existence of an invasive bridgehead effect at a worldwide scale [9]. Native to Asia, HA has been introduced repeatedly as a biocontrol agent against aphids since 1916 in North America [10], [11], since 1982 in Europe [12] and since 1986 in South America [13]. Despite recurrent intentional releases of ladybirds originating from various source populations in its native range for acclimation attempts, the species did not establish for decades. However, for unknown reasons it recently and suddenly became invasive on four different continents. Invasive populations were first recorded in eastern (Louisiana, USA) and western (Oregon, USA) North America in 1988 and 1991, respectively [14], [15]. They were then recorded in Europe (Belgium) [16] and South America (Argentina) [17] in 2001 and in Africa (South Africa) [18] in 2004. The species has spread widely in these areas where it has become a harmful predator of non-target arthropods, a household invader, and a pest of fruit production [19].

Most of our knowledge about introduction pathways of invasive species relies on historical and observational data, which are often sparse, incomplete and sometimes misleading. Population genetics has proven to be a useful approach to reconstruct routes of introduction, highlighting how complex and counter-intuitive the real story can be [4], [5]. This is especially true since the recent development of a new approach termed approximate Bayesian computation (ABC) [20], [21], [22], [23], [24]. Using molecular and historical data, this method allows performing model-based inference in a Bayesian setting for complex demographic or evolutionary scenarios such as those related to the introduction histories of invasive species, where bottleneck, multiple introductions and/or genetic admixture events are often suspected. Here, we retraced the routes of all five worldwide invasive HA populations using a rigorous quantitative analysis of microsatellite genetic variation relying on ABC methods. We compared large sets of HA invasion scenarios covering all invaded areas, taking into account historical data (e.g. dates of first observation of the outbreaks and dates of initial collection of biocontrol strains) and all potential sources (native, older outbreaks and biocontrol), accounting for the possibility of genetic admixture among them.

## Results and Discussion

**Figure 1:**
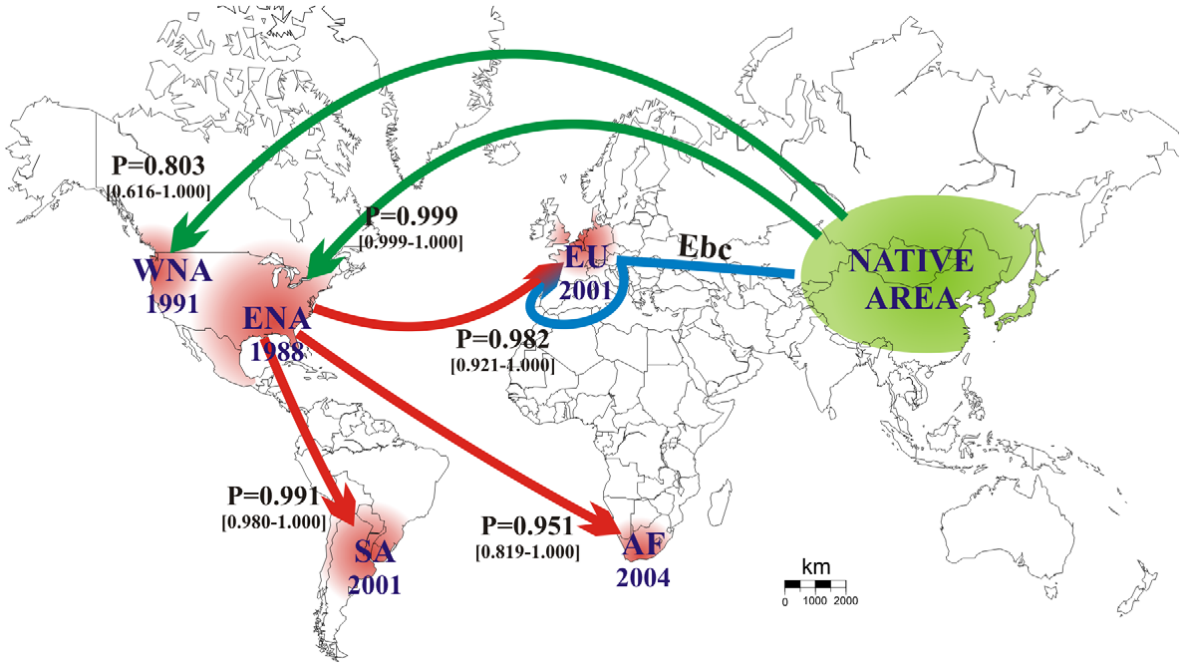
Worldwide routes of invasion of *Harmonia axyridis*. Most likely scenario of invasions into eastern North America (ENA), western North America (WNA), South America (SA), Europe (EU) and Africa (AF) by *Harmonia axyridis*, deduced from analyses based on approximate Bayesian computation. For each outbreak, the arrow indicates the most likely invasion pathway and the associated posterior probability value (P), with 95% confidence intervals in brackets. Years of first observation of invasive populations are indicated. Initially collected from the native area in 1982, the European biocontrol strain (Ebc; blue arrow) was used for biocontrol efforts in Europe and South America. Introductions to North America from the native area (green arrows) may have involved releases for biocontrol efforts.

**Figure 2:**
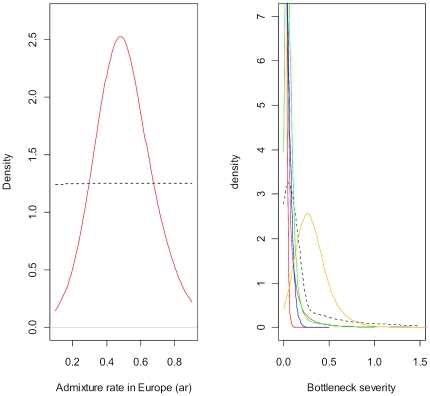
Posterior distributions of the genetic admixture rate in Europe (left panel) and the bottleneck severity for the five outbreaks (right panel) of *Harmonia axyridis*. The best estimates of admixture and bottleneck severity occur where the posterior probability density function peaks. Left panel: admixture in Europe involves the European biocontrol strain at a rate ar and the eastern North American population at a rate 1-ar. Y-axis: probability density of the genetic admixture ar in Europe. The dotted line is the prior distribution of admixture rate. Right panel: bottleneck severity was computed as the ratio between the duration (in number of generations) of the bottleneck following introduction and the effective number of individuals during this period [28], [29]. Y-axis: probability density of bottleneck severity. Continuous lines in red, blue, maroon, green and orange are the posterior distributions of the European, eastern North American, western North American, South African and South American outbreaks, respectively. The dotted line is the prior distribution of bottleneck severity. It ranges from zero (complete absence of bottleneck) to 2.5 (strong bottleneck; i.e. 2 effective individuals during 5 generations). Posterior distributions support bottleneck severity values considerably lower than those of the prior, except for South America.

Our results unambiguously point to a surprising scenario (Fig. 1). The outbreaks in eastern and western North America originated from two independent introductions from the native range (either from biocontrol or accidental introductions). The South American and African outbreaks both originated independently from eastern North America. The European outbreak also originated from eastern North America, but with substantial genetic admixture with individuals of the European biocontrol strain (estimated at 43%, 95%CI: [18%–83%]; Fig. 2a). The establishment of new invasive populations is characterized by low (South America) to minute (all other outbreaks) bottleneck severities suggesting a substantial number of founding individuals and/or a quick demographic recovery (Fig. 2b). Each choice of scenario is supported by very high posterior probabilities using two different sets of prior distributions of demographic, historic and mutation parameters (Fig. 1, Table S2 and Table S3). In all analyses the 95% CI of the most likely scenario never overlapped with those of competing scenarios. We also computed the type I and type II errors from control simulated data sets for each of the five nested analyses and found that our method selected the true scenario with high confidence and markedly low type II errors (Table 1).

**Table 1:** Confidence in scenario selection obtained from the ABC analyses.

Invaded area (ABC analysis)	Number of competing scenarios	Selected scenario	Type I error	Type II error Mean (min – max)
Eastern North America (Analysis 1)	3	Introduction from the native area	0.12	0.080 (0.03–0.13)
Western North America (Analysis 2)	6	Introduction from the native area	0.16	0.030 (0.00–0.12)
Europe (Analysis 3)	10	Admixture between eastern North America and European biocontrol	0.16	0.010 (0.00–0.04)
South America (Analysis 4)	10	Introduction from eastern North America	0.03	0.013 (0.00–0.06)
Africa (Analysis 5)	21	Introduction from eastern North America	0.12	0.006 (0.00–0.06)

For the scenarios not complicated by substantial admixture, the raw classical statistics measuring genetic variation between populations such as pairwise *F*_ST_ and assignment likelihood [23] agree with our conclusions based on ABC methods (Table S4). On the other hand, such classical statistics are unable to detect North America and the European biocontrol strain as the sources of the admixed European invasive population. Rather, these simpler methods suggest that the latter outbreak originated from the native area only. We show with simulated data that, in case of admixture between two populations deriving from the same source, pairwise *F*_ST_ and assignment likelihood values tend to select the ancestral population (here the native area) as the source of the admixed population (Fig. S2). An admixed origin of the European invasive population involving eastern North America and the European biocontrol strain as inferred from ABC is also supported by the raw allelic distributions observed in these samples (see Fig. S3 for an illustration). The ABC methods used here have hence three advantages over more standard methods based on raw measures of genetic distance: they use all the data simultaneously in inference, allow an assessment of uncertainty in all inferences and therefore provide confidence in the choice of invasion routes [23], and they avoid misleading biases such as those due to genetic admixture (if included in the set of compared scenarios), an increasingly acknowledged common feature of species invasions [3], [5].

The complete panorama of the invasion history of HA strikingly points to the worldwide dissemination of a single successful invasive population (eastern North America) into remote new territories. Although ladybirds were repeatedly introduced in North America, Europe and South America for biocontrol from native Asian populations and laboratory strains, eastern North America is the proximate origin of the worldwide invasion of HA. This pattern illustrates what we call the invasive bridgehead effect, whereby a particular invasive population serves as the source of colonists for other areas. Our results have important implications for the understanding and the management of biological invasions. The history of HA reveals that prevention of new invasions should include minimizing accidental dissemination from invasive bridgehead populations (here eastern North America) rather than focusing on the native range of invasive species. For instance, we found that live HA individuals intercepted in Europe (Norway) in 2007 on imported timber most likely originated from eastern North America based on the sample being significantly differentiated genetically from all tested populations (*p*<1×10^−6^) except that of eastern North America (*p* = 0.060). The implication of eastern North America being an invasive bridgehead, together with the absence of remote establishment and slower local spread of the population in western North America and the long history of unsuccessful biocontrol introductions of HA from its native range, jointly suggest that an evolutionary shift triggering invasion occurred in eastern North America. It is worth pointing out that the invasive bridgehead effect is evolutionarily parsimonious: a single evolutionary shift in a single introduced population (the bridgehead) is required whereas multiple changes are required if introduced populations had become invasive independently. In Europe, the potential role of admixture with the European biocontrol strain is unknown, but the single eastern North American origin of the South American and South African outbreaks suggests that the genetic admixture observed in Europe is not required for an eastern North American propagule to establish and start an invasive population in diverse ecological contexts.

In conclusion, our results provide a convincing demonstration of an invasive bridgehead scenario in a worldwide emblematic invasive species. That invasion can proceed via a bridgehead has important implications both for invasion theory (i.e. the number and nature of evolutionary shift(s) involved in large scale invasions) and for ongoing efforts to manage invasions by alien organisms (i.e. heightened vigilance against invasive bridgehead populations). Our study highlights the interest of new model-based methods such as approximate Bayesian computation to rigorously compare complex invasion scenarios, including those with genetic admixture, a feature of species invasions that is increasingly acknowledged to be common [2], [3], [5]. It would be useful to revisit previously published genetic data sets on other worldwide invasive species using similar ABC approaches to examine whether bridgeheads and/or admixtures are a common mechanism driving invasion. Our ability to confidently reconstruct the routes of introduction of worldwide invaders is crucial to generating sensible hypotheses regarding the environmental and evolutionary factors responsible for biological invasions [3]. In HA, our results suggest that an evolutionary shift triggering invasion likely occurred in the bridgehead population in eastern North America. Forthcoming studies based on quantitative genetics approaches [3], [25] will shade light on the evolutionary basis of HA invasiveness in eastern North America and into the potential role of genetic admixture in Europe.

## Materials and Methods

### Population samples and genotyping

Population samples were genotyped at 18 microsatellite markers [26]. The samples of populations from the five invaded areas (Fig. 1 and Table S1) were collected near the sites where the outbreaks were first observed. The three samples from the native range were collected in eastern Asia, where individuals historically used for biocontrol purposes had been collected [10], [11], [12]. The three native population samples were genetically homogeneous (mean pairwise *F*_ST_ = 0.0057), and were therefore pooled into a single sample. Using native population samples individually did not change our conclusions. We genotyped numerous additional population samples collected from all invaded areas (30 additional samples) and from the native range (5 additional samples); analysis of these samples indicated that the subset of samples used in the study provides an adequate description of the main invasive and native populations (i.e. none significant to low level of genetic differentiation within the native range and within each invaded area). The European biocontrol sample (Ebc) was from the INRA rearing stock of 1987 initially collected from the native area in 1982 and used for biocontrol in Europe and South America. Genotyping of the biocontrol strains produced in European biofactories from the late 90's to the present (4 additional samples) confirmed that they are indeed derived from the original INRA strain we genotyped.

### Inferring invasion scenarios using approximate Bayesian computation

Genetic variation within and between populations was summarized using a set of statistics traditionally employed in approximate Bayesian computation analyses (ABC) [23], [24]. For each population and each population pair we used the mean number of alleles per locus, the mean expected heterozygosity and the mean allelic size variance. The other statistics used were the mean ratio of the number of alleles over the range of allele sizes, pairwise *F*_ST_ values, mean individual assignment likelihoods of population *i* assigned to population *j* and the maximum likelihood estimate of admixture proportion.

We performed five serial nested ABC analyses of invasion scenarios involving successive HA outbreaks (Table S1). In analysis 1, we dealt with the introduction pathway for the first recorded outbreak in eastern North America in 1988, with native populations (either from biocontrol or accidental introduction) and/or the European biocontrol population as potential sources, thereby defining three competing scenarios (see Fig. S1). The second outbreak in western North America in 1991 was examined in analysis 2, taking into account the scenario selected in analysis 1. For this analysis, there were three possible sources: the first outbreak (eastern North America), the native range, and the European biological control strain (Table S1). With the potential for admixture between them, this gave 6 competing scenarios. The European and South American outbreaks in 2001 were addressed in analyses 3 and 4, respectively (10 scenarios for each outbreak), taking into account the scenario selected in analysis 1 and 2. The year of first observation in Europe and South America was the same, thus we assumed that one could not be a potential source of the other. Nevertheless, we performed a specific ABC analysis to test this assumption which confirmed the independence of these two outbreaks (P = 0.773 with CI = 0.555–0.991). Finally, the African outbreak in 2004 was considered in analysis 5 (21 scenarios), taking into account the scenarios selected in analyses 1, 2, 3 and 4.

The ABC analyses were performed using parameter values drawn from the prior distributions described in Table S2 (prior set 1), and by simulating 10^6^ microsatellite data sets for each competing scenario in the first four analyses and 5×10^5^ data sets in analysis 5 because of the high number of scenarios (21) and summary statistics (170) which made a larger analysis computationally unfeasible. For each of the five analyses, we estimated the posterior probabilities of the competing scenarios using a polychotomous logistic regression [24] on the 0.1% of simulated data sets closest to the observed data set. The selected scenario is that with the highest significant probability value with a non-overlapping 95% confidence interval. We estimated the posterior distributions of demographic parameters under the final HA invasion scenario presented in Fig. 1 using a local linear regression on the 1% closest of 5×10^6^ simulated data sets [22], [24].

### Confidence in scenario selection

We evaluated the robustness of our inferences by running a second analysis with an alternative set of priors (prior set 2 in Table S2) and by estimating the posterior probabilities of scenarios using the 0.1% or 1% closest simulated data sets for both sets of priors (Table S2). For each of our five ABC analyses, we evaluated the ability of the methodology to correctly select the true scenario by analyzing test data sets simulated from the various competing scenarios with the same number of loci and individuals as in the real data set. For each scenario, one hundred such data sets were simulated using parameter values drawn from the same probability distributions as the priors (prior set 1, Table S2). Posterior probabilities of each competing scenario were estimated for each simulated test data set using the 0.1% closest data sets. These probabilities were used to compute type I and II errors in the selection of scenarios.

Most ABC computations were processed using a modified version of the Windows package DIYABC [24]. Some of the computations for assessing confidence in scenario selection (i.e. those for analysis 5) necessitated the development of specific Linux programs and scripts running on a computer cluster (available under request from A.E.).

### Additional simulation and statistical treatments

We ran computer simulations to tackle the question of a high frequency of erroneous selection of the source population in a situation of genetic admixture when considering only the raw values of genetic differentiation statistics (i.e. pairwise *F*_st_ and assignment likelihood values). We used the program DIYABC to simulate genetic data sets under a scenario of invasion with admixture similar to the one considered in the case of the invasion of Europe by HA. We simulated 6×10^4^ data sets drawing the parameter values in the prior set 1 (Table S2), except the admixture rate (*ar*) which was drawn in a discrete {0, 0.1, …,1.0} distribution instead of a uniform [0.1;0.9] distribution. The number of sampled diploid individuals was fixed to 30 in all populations. The deduced origin of the admixed introduced population is the population with which the *F*_ST_-value is the smallest or the assignment likelihood value is the highest. We then calculated the proportion of simulated data sets, as a function of the admixture rate, for which the deduced origin was the ancestral population of the two actual source populations (i.e. the native area in the case of the invasion of Europe by HA), considering either *F*_ST_ or assignment likelihood values (see Fig. S2).

Finally, we also genotyped 200 live HA individuals intercepted in Europe (Åndalsnes, Norway) in 2007 on timber imported from North America. We tested for the genetic differentiation, using Fisher's exact tests [27], between this interception HA sample and each population sample used in the ABC analysis to elucidate its most likely origin.

## Supporting Information

Figure S1 Graphic representation of the three competing HA invasion scenarios considered in ABC analysis 1, which focused on the origin of the eastern North American outbreak ENA. Notes: Time 0 is the sampling year 2007 and time 50 is the sampling year 1987 (2.5 generations per year). Pop 1 is the HA population from the native area; Pop 2 is the European biocontrol strain (EBC); Pop 3 is the eastern North American population (ENA); Population 4 (light blue segment) corresponds to an unsampled biocontrol strain released in eastern North America. Introduction events in the wild include a period of *BD* generation(s) of potentially small population size (*NF_3_* for pop 3). Scenario 1 corresponds to a native origin of the ENA outbreak, possibly through an intermediate biocontrol population (population 4). Scenario 2 corresponds to a European Biocontrol origin of the ENA outbreak. In scenario 3, the ENA outbreak is the result of an admixture between individuals from the native area at a rate *ar* and from the European biocontrol strain at a rate 1-*ar*. All parameters with associated prior distributions are described in Table S2.
(5.94 MB TIF)

Figure S2 Erroneous selection of source population in a situation of genetic admixture when using raw values of genetic differentiation statistics. Notes: (a) We used the program DIYABC [24] to simulate genetic data sets under a scenario of invasion with admixture similar to the one considered in the case of the invasion of Europe by HA. The two sources populations Pop 2 and Pop 3 of the admixed population Pop 4 derive from the same population Pop 1. Pop 1 stands for the HA population from the native area, Pop 2 for the European biocontrol strain (EBC), Pop 3 for the eastern North American population (ENA), and Pop 4 for the European population (EU). Population 5 (light blue segment) corresponds to an unsampled biocontrol strain released in eastern North America. *NS*_k_ stand for the stable effective population sizes in population k. Introduction events in the wild include a period of *BD* generation(s) of potentially small population size (*NF_3_* for Pop 3 and *NF_4_* for Pop 4). We simulated 6×10^4^ data sets drawing the parameter values in the prior set 1 (Table S2), except the admixture rate (ar) which was drawn in a discrete {0, 0.1, …,1.0} distribution instead of a uniform [0.1;0.9] distribution. (b) Pairwise *F*_st_ and assignment likelihood values were computed for each simulated data set between the (admixed) Pop 4 and other populations. The deduced origin of Pop 4 is the population with which its *F*_st_-value is the smallest or its assignment likelihood value is the highest. We have represented here the proportion of simulated data sets, as a function of the admixture rate, for which the deduced origin was the ancestral population of the two actual source populations (i.e., Pop 1), considering either *F*_st_ or assignment likelihood values.
(7.30 MB TIF)

Figure S3 Raw signatures of admixture in the HA invasive population in Europe. Notes: We present here the histograms of allele frequencies at two of the 18 microsatellite loci genotyped in the invasive populations from Europe (EU) and Eastern North America (ENA), and from the European biocontrol strain (Ebc). Only a mixture of the Eastern North American and biocontrol gene pools makes it possible to generate all alleles observed in the European invasive population.
(9.97 MB TIF)

Table S1 Native, invasive and biocontrol populations of *Harmonia axyridis* (HA), with the possible sources of each population considered for each nested ABC analysis. Notes: The definition of potential sources is based on the year of first observation of invasive populations. The EBC population was considered a potential source in all analyses even though historical records indicate that it was used for biocontrol purposes only in Europe and South America [13]. Admixtures between all pairs of potential sources also were considered in specific scenarios. In analyses 1 and 2, when the native population was involved we considered that there may have been a period of laboratory rearing in preparation for release for biocontrol purposes resulting in a lower effective population size, instead of a direct introduction from the native area (see Table S2 and Figure S1). For each ABC analysis, the number of competing scenarios is given in parentheses. Population code names as in Figure 1.
(0.06 MB DOC)

Table S2 Two sets of prior distributions of demographic, historic and mutation parameters used in ABC analyses. Notes: Populations *i* are wild populations (invasive and native) and populations *k* are biocontrol strains (i.e., laboratory reared populations). Times were translated into numbers of generations running back in time from 2007 by assuming 2.5 generations per year in prior set 1, and 3 generations per year in prior set 2 [19]. *NS* = stable effective population size (number of diploid individuals); *NF* = effective number of founders during an introduction step lasting *BD* generation(s); *ar* = admixture rate (only for scenarios with admixture); *t_i_* = introduction date of invasive populations *i* with bounds *x_i_* or *y_i_* fixed from dates of first observation, assuming 2.5 or 3 generations per year, respectively; *tbc* = creation date of unsampled biocontrol strain for ENA and WNA populations (with condition *t_i_*<or = *tbc_i_*) bounded between the dates of first observation of invasive population (which would correspond to a direct introduction into the wild) and the number of generations from 1970, the start date of a period of intense HA biocontrol activity in the USA. For microsatellite marker parameters, the loci were assumed to follow a generalized stepwise mutation model [S1] with two parameters: the mean mutation rate (mean *μ*) and the mean parameter of the geometric distribution (mean *P*) of the length in number of repeats of mutation events. Each locus has a possible range of 40 contiguous allelic states and is characterized by individual *μ_loc_* and *P_loc_* values, with *μ_loc_* drawn from a Gamma (mean = mean *μ* and shape = 2) distribution and *P_loc_* drawn from a Gamma (mean = mean P and shape = 2) distribution [S2]. Uneven insertion/deletion events that were suspected for several of our microsatellite loci based on observed allele sizes (i.e., allele lengths were sometimes not multiple of the motif length implying that there has been insertion-deletion mutations [28]) were also simulated with a mean mutation rate *μ*SNI (for single nucleotide instability) and *μ*SNI_loc_ drawn for each locus from a Gamma (mean = mean *μ*SNI and shape = 2). Boundaries of distributions are in brackets. Parameters of Normal and Gamma distributions are in parentheses. In prior set 2, Normal, Lognormal and Gamma distributions are truncated between the same boundaries as in prior set 1. All prior quantities presented were computed from 100,000 values. NA = not applicable; DV = can take different values. Supporting references: S1. Estoup A, Jarne P, Cornuet JM (2002) Homoplasy and mutation model at microsatellite loci and their consequences for population genetics analysis. Molecular Ecology 11: 1591–1604. S2. Verdu P, Austerlitz F, Estoup A, Vitalis R, Georges M, et al. (2009) Origins and Genetic Diversity of Pygmy Hunter-Gatherers from Western Central Africa. Current Biology 19: 1–7.
(0.07 MB DOC)

Table S3 Posterior probabilities (P) of the selected (most likely) scenarios in each ABC analysis at two different thresholds of smallest Euclidian distances (0.1% and 1%) and two different sets of priors. Notes: Prior sets are detailed in Table S2. 95% confidence intervals (CI) are in brackets. The 95% CI of the selected scenarios never overlapped those of competing scenarios. The values presented in Figure 1 of the main text are those obtained using the 0.1% threshold and prior set 1.
(0.05 MB DOC)

Table S4 Classical population genetics statistics of the studied HA populations and inferred source populations of the five HA invasive outbreaks. Notes: Mean corrected number of alleles per locus (*Na*), expected heterozygosity (*He*) [S3], pairwise *F*_ST_ matrix and mean individual assignment log-likelihood of invasive populations to putative source populations (in parentheses) [28]. Dashes refer to the pairs of invasive and source populations that are chronologically incompatible. The deduced origin of each outbreak is the sample for which the *F*_ST_-value is the smallest and the assignment likelihood is maximized (values in bold). Ebc = European biocontrol strain; ENA = Eastern North America; WNA = Western North America; SA = South America; EU = Europe; AF = Africa. Supporting reference: S3. Nei M (1978) Estimation of average heterozygosity and genetic distance from a small number of individuals. Genetics 89: 583–590.
(0.06 MB DOC)

## References

[1] Puth LM, Post DM (2005). Studying invasion: have we missed the boat?. Ecology Letters.

[2] Wilson JRU, Dormontt EE, Prentis PJ, Lowe AJ, Richardson DM (2009). Something in the way you move: dispersal pathways affect invasion success.. Trends in Ecology & Evolution.

[3] Keller SR, Taylor DR (2008). History, chance and adaptation during biological invasion: separating stochastic phenotypic evolution from response to selection.. Ecology Letters.

[4] Miller N, Estoup A, Toepfer S, Bourguet D, Lapchin L (2005). Multiple transatlantic introductions of the western corn rootworm.. Science.

[5] Kolbe JJ, Glor RE, Schettino LRG, Lara AC, Larson A (2004). Genetic variation increases during biological invasion by a Cuban lizard.. Nature.

[6] Downie DA (2002). Locating the sources of an invasive pest, grape phylloxera, using a mitochondrial DNA gene genealogy.. Molecular Ecology.

[7] Floerl O, Inglis GJ, Dey K, Smith A (2009). The importance of transport hubs in stepping-stone invasions.. Journal of Applied Ecology.

[8] Hanfling B, Carvalho GR, Brandl R (2002). mt-DNA sequences and possible invasion pathways of the Chinese mitten crab.. Marine Ecology-Progress Series.

[9] Vilà M, Basnou C, Gollasch S, Josefsson M, Pergl J, DAISIE, editor (2009). One Hundred of the Most Invasive Alien Species in Europe.. Handbook of Alien Species in Europe: Springer Netherlands.

[10] Krafsur ES, Kring TJ, Miller JC, Nariboli P, Obrycki JJ (1997). Gene flow in the exotic colonizing ladybeetle Harmonia axyridis in North America.. Biological Control.

[11] Tedders WL, Schaefer PW (1994). Release and Establishment of *Harmonia Axyridis* (Coleoptera, Coccinellidae) in the Southeastern United-States.. Entomological News.

[12] Ongagna P, Giuge L, Iperti G, Ferran A (1993). Life-cycle of *Harmonia axyridis* (Col, Coccinellidae) in its area of introduction - South-Eastern France.. Entomophaga.

[13] Poutsma J, Loomans AJM, Aukema B, Heijerman T (2008). Predicting the potential geographical distribution of the harlequin ladybird, Harmonia axyridis, using the CLIMEX model.. Biocontrol.

[14] Chapin J, Brou V (1991). *Harmonia axyridis* (Pallas), the third species of the genus to be found in the United States (Coleoptera: Coccinellidae).. Proceedings of the Entomological Society of Washington.

[15] LaMana ML, Miller JC (1996). Field observations on Harmonia axyridis Pallas (Coleoptera: Coccinellidae) in Oregon.. Biological Control.

[16] Adriaens T, Branquart E, Maes D (2003). The Multicoloured Asian Ladybird *Harmonia axyridis* Pallas (Coleoptera : Coccinellidae), a threat for native aphid predators in Belgium?. Belgian Journal of Zoology.

[17] Saini E (2004). Presencia de *Harmonia axyridis* (Pallas) (Coleoptera : coccinellidae) en la provincia de Buenos aires. Aspectos biologicos y morfologicos.. RIA.

[18] Stals R, Prinsloo G (2007). Discovery of an alien invasive, predatory insect in South Africa: the multicoloured Asian ladybird beetle, Harmonia axyridis (Pallas) (Coleoptera: Coccinellidae).. South African Journal of Science.

[19] Koch RL (2003). The multicolored Asian lady beetle, *Harmonia axyridis*: A review of its biology, uses in biological control, and non-target impacts.. Journal of Insect Science.

[20] Tavare S, Balding DJ, Griffiths RC, Donnelly P (1997). Inferring coalescence times from DNA sequence data.. Genetics.

[21] Pritchard JK, Seielstad MT, Perez-Lezaun A, Feldman MW (1999). Population growth of human Y chromosomes: A study of Y chromosome microsatellites.. Molecular Biology and Evolution.

[22] Beaumont MA, Zhang WY, Balding DJ (2002). Approximate Bayesian computation in population genetics.. Genetics.

[23] Guillemaud T, Beaumont MA, Ciosi M, Cornuet JM, Estoup A (2010). Inferring introduction routes of invasive species using approximate Bayesian computation on microsatellite data.. Heredity.

[24] Cornuet JM, Santos F, Beaumont MA, Robert CP, Marin JM (2008). Inferring population history with DIY ABC: a user-friendly approach to approximate Bayesian computation.. Bioinformatics.

[25] Facon B, Jarne P, Pointier JP, David P (2005). Hybridization and invasiveness in the freshwater snail *Melanoides tuberculata*: hybrid vigour is more important than increase in genetic variance.. Journal of Evolutionary Biology.

[26] Loiseau A, Malausa T, Lombaert E, Martin JF, Estoup A (2009). Isolation and characterization of microsatellites in the harlequin ladybird, *Harmonia axyridis* (Coleoptera, Coccinellidae), and cross-species amplification within the family Coccinellidae.. Molecular Ecology Resources.

[27] Raymond M, Rousset F (1995). Genepop (version. 1.2), a population genetics software for exact tests and ecumenicism.. Journal of Heredity.

[28] Pascual M, Chapuis MP, Mestres F, Balanya J, Huey RB (2007). Introduction history of Drosophila subobscura in the New World: a microsatellite-based survey using ABC methods.. Molecular Ecology.

[29] Wright SI, Bi IV, Schroeder SG, Yamasaki M, Doebley JF (2005). The effects of artificial selection on the maize genome.. Science.

