# Supplementary figures and images for: Bridgehead Effect in the Worldwide Invasion of the Biocontrol Harlequin Ladybird
Source: PLoS One. 2010 Mar 17;5(3):e9743. doi: 10.1371/journal.pone.0009743 (PMC2840033; doi:10.1371/journal.pone.0009743)

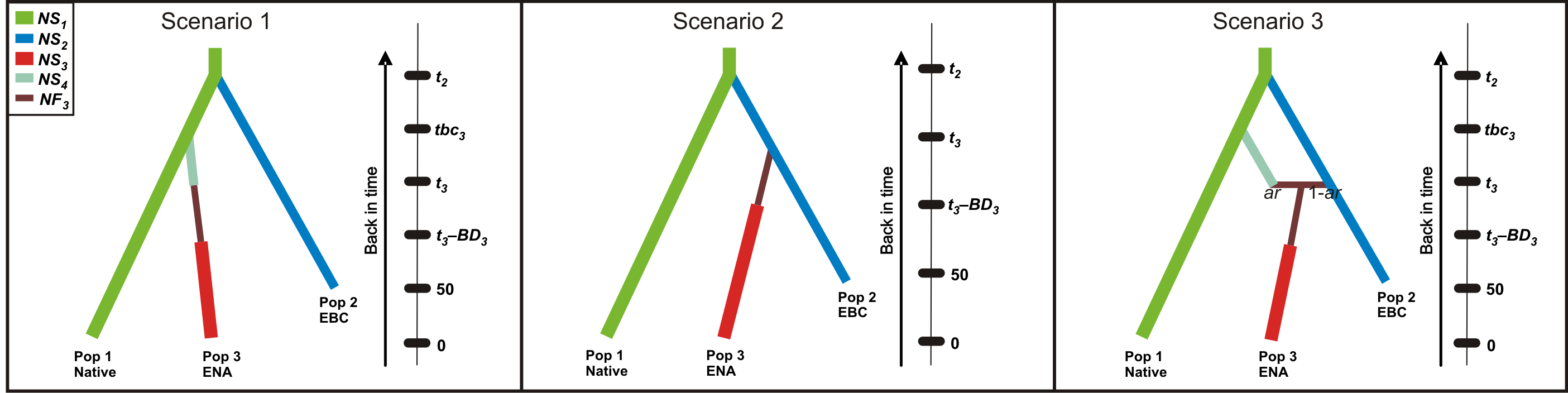

Supplement: Figure S1 — Graphic representation of the three competing HA invasion scenarios considered in ABC analysis 1, which focused on the origin of the eastern North American outbreak ENA. Notes: Time 0 is the sampling year 2007 and time 50 is the sampling year 1987 (2.5 generations per year). Pop 1 is the HA population from the native area; Pop 2 is the European biocontrol strain (EBC); Pop 3 is the eastern North American population (ENA); Population 4 (light blue segment) corresponds to an unsampled biocontrol strain released in eastern North America. Introduction events in the wild include a period of BD generation(s) of potentially small population size (NF3 for pop 3). Scenario 1 corresponds to a native origin of the ENA outbreak, possibly through an intermediate biocontrol population (population 4). Scenario 2 corresponds to a European Biocontrol origin of the ENA outbreak. In scenario 3, the ENA outbreak is the result of an admixture between individuals from the native area at a rate ar and from the European biocontrol strain at a rate 1-ar. All parameters with associated prior distributions are described in Table S2. (5.94 MB TIF) [file pone.0009743.s001.tif]

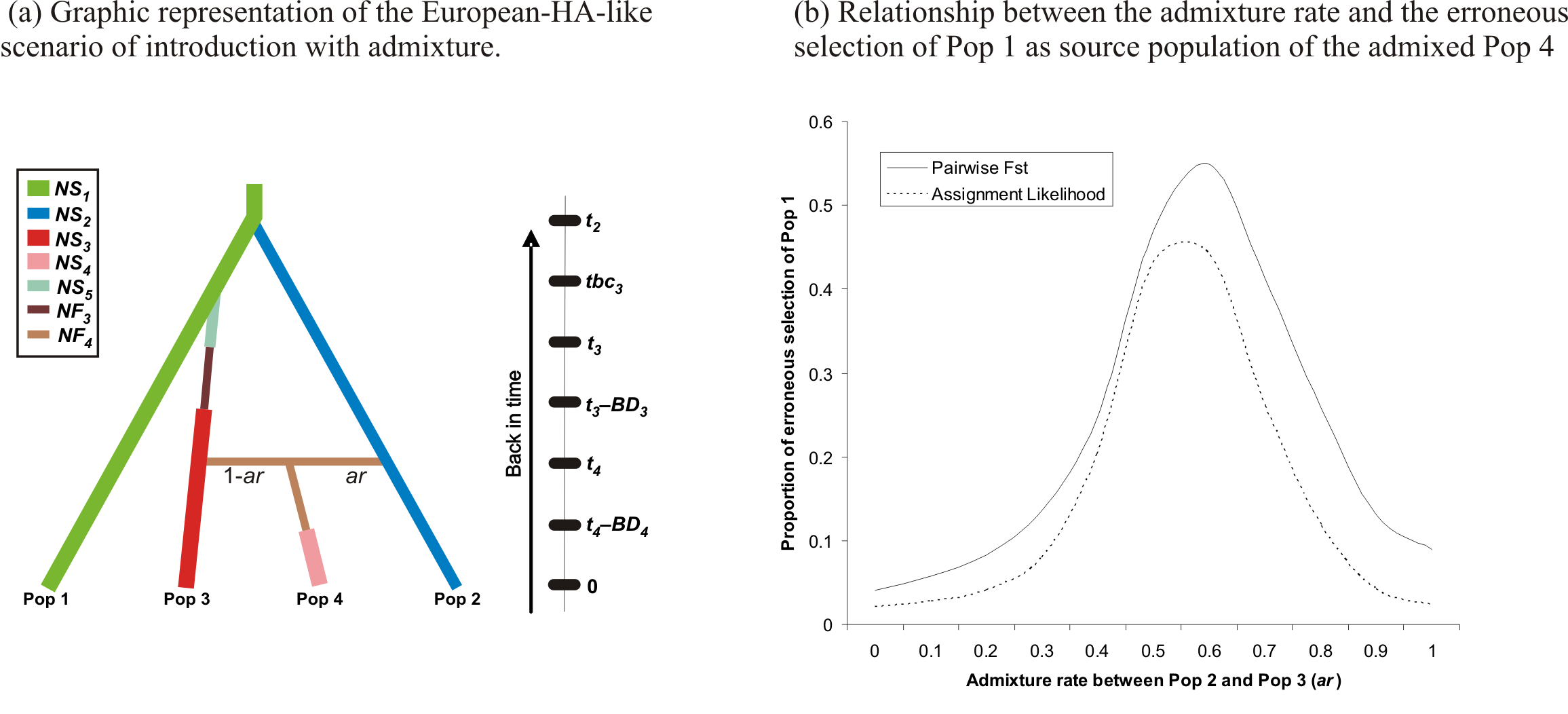

Supplement: Figure S2 — Erroneous selection of source population in a situation of genetic admixture when using raw values of genetic differentiation statistics. Notes: (a) We used the program DIYABC [24] to simulate genetic data sets under a scenario of invasion with admixture similar to the one considered in the case of the invasion of Europe by HA. The two sources populations Pop 2 and Pop 3 of the admixed population Pop 4 derive from the same population Pop 1. Pop 1 stands for the HA population from the native area, Pop 2 for the European biocontrol strain (EBC), Pop 3 for the eastern North American population (ENA), and Pop 4 for the European population (EU). Population 5 (light blue segment) corresponds to an unsampled biocontrol strain released in eastern North America. NS k stand for the stable effective population sizes in population k. Introduction events in the wild include a period of BD generation(s) of potentially small population size (NF3 for Pop 3 and NF4 for Pop 4). We simulated 6×10^4^ data sets drawing the parameter values in the prior set 1 (Table S2), except the admixture rate (ar) which was drawn in a discrete {0, 0.1, …,1.0} distribution instead of a uniform [0.1;0.9] distribution. (b) Pairwise F st and assignment likelihood values were computed for each simulated data set between the (admixed) Pop 4 and other populations. The deduced origin of Pop 4 is the population with which its F st-value is the smallest or its assignment likelihood value is the highest. We have represented here the proportion of simulated data sets, as a function of the admixture rate, for which the deduced origin was the ancestral population of the two actual source populations (i.e., Pop 1), considering either F st or assignment likelihood values. (7.30 MB TIF) [file pone.0009743.s002.tif]

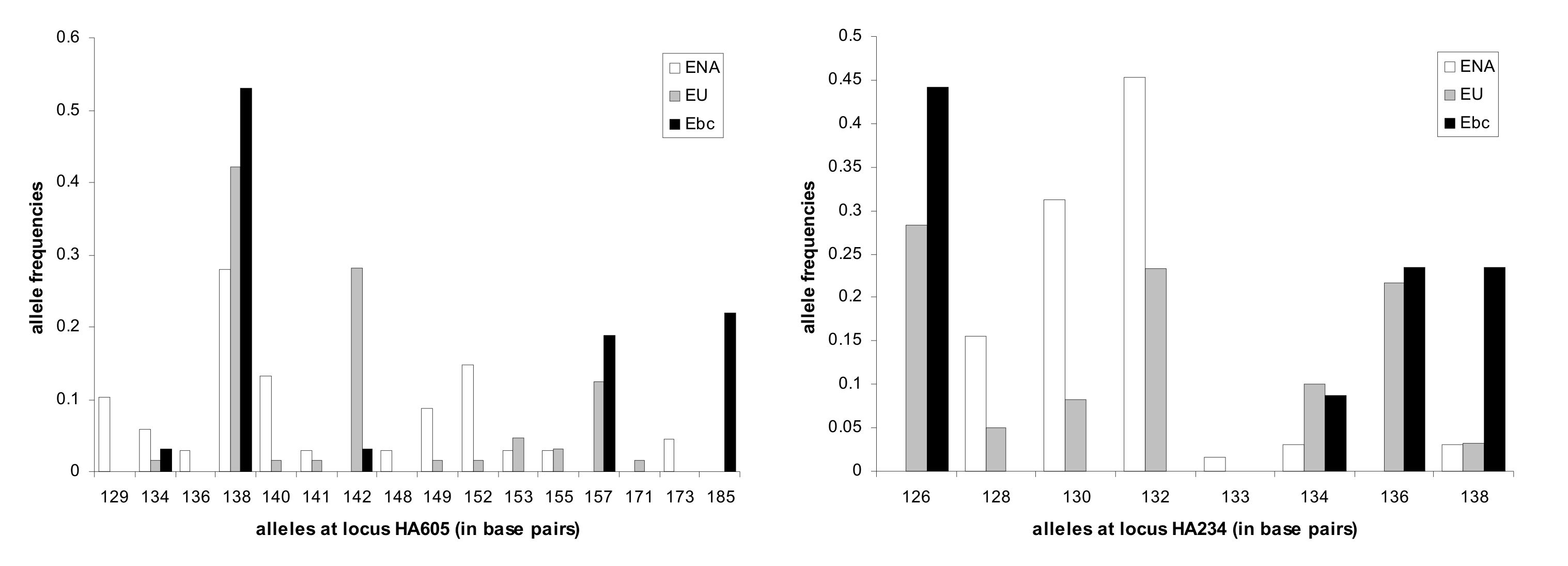

Supplement: Figure S3 — Raw signatures of admixture in the HA invasive population in Europe. Notes: We present here the histograms of allele frequencies at two of the 18 microsatellite loci genotyped in the invasive populations from Europe (EU) and Eastern North America (ENA), and from the European biocontrol strain (Ebc). Only a mixture of the Eastern North American and biocontrol gene pools makes it possible to generate all alleles observed in the European invasive population. (9.97 MB TIF) [file pone.0009743.s003.tif]
