# Supplementary material for: Bridgehead Effect in the Worldwide Invasion of the Biocontrol Harlequin Ladybird
Source: PLoS One. 2010 Mar 17;5(3):e9743. doi: 10.1371/journal.pone.0009743 (PMC2840033; doi:10.1371/journal.pone.0009743)
Supplement: Table S1 — Native, invasive and biocontrol populations of Harmonia axyridis (HA), with the possible sources of each population considered for each nested ABC analysis. Notes: The definition of potential sources is based on the year of first observation of invasive populations. The EBC population was considered a potential source in all analyses even though historical records indicate that it was used for biocontrol purposes only in Europe and South America [13]. Admixtures between all pairs of potential sources also were considered in specific scenarios. In analyses 1 and 2, when the native population was involved we considered that there may have been a period of laboratory rearing in preparation for release for biocontrol purposes resulting in a lower effective population size, instead of a direct introduction from the native area (see Table S2 and Figure S1). For each ABC analysis, the number of competing scenarios is given in parentheses. Population code names as in Figure 1. (0.06 MB DOC) [file pone.0009743.s004.doc]

**Table S1**

| Population  (code name) | Date of 1^st^ observation | Sampling  location | Geographic  coordinates | sampling  date | Number of genotyped individuals | Potential sources | ABC Analyses |
| --- | --- | --- | --- | --- | --- | --- | --- |
| Native 1 | - | Beijing, China | 40.24°N  116.23°E | May  2007 | 28 | - | - |
| Native 2 | - | Shilin city, Yunnan, China | 24.90°N  103.35°E | August  2007 | 35 | - | - |
| Native 3 | - | Fuchu, Japan | 34.57°N  133.24°E | September  2005 | 36 | - | - |
| European Biocontrol (EBC) | 1982 [12]  In laboratory | Rearing stock, INRA laboratory | - | April  1987 | 18 | Native | - |
| Eastern North-America (ENA) | 1988 [14] | Joyce, Louisiana, USA | 31.94°N  92.60°W | November  2007 | 34 | Native (possibly through a biocontrol release); EBC; admixture between both potential sources | Analysis 1  (3 scenarios) |
| Western North-America (WNA) | 1991 [15] | Sunnyside, Washington, USA | 46.32°N  120.01°W | September  2007 | 42 | Native (possibly through a biocontrol release); EBC; ENA; admixture between all pairs of potential sources | Analysis 2  (6 scenarios) |
| Europe (EU) | 2001 [16] | Gent, Belgium | 51.05°N  3.71°E | October  2007 | 32 | Native; EBC; ENA; WNA; admixtures between all pairs of potential sources | Analysis 3  (10 scenarios) |
| South America (SA) | 2001 [17] | Curitiba, Brazil | 25.45°S  49.24°W | February  2008 | 30 | Native; EBC; ENA; WNA; admixtures between all pairs of potential sources | Analysis 4  (10 scenarios) |
| Africa (AF) | 2004 [18] | Somerset West, South Africa | 34.03°S  18.83°E | May  2008 | 31 | Native; EBC; ENA; WNA; EU; SA; admixtures between all pairs of potential sources | Analysis 5  (21 scenarios) |
