# Supplementary material for: Bridgehead Effect in the Worldwide Invasion of the Biocontrol Harlequin Ladybird
Source: PLoS One. 2010 Mar 17;5(3):e9743. doi: 10.1371/journal.pone.0009743 (PMC2840033; doi:10.1371/journal.pone.0009743)
Supplement: Table S2 — Two sets of prior distributions of demographic, historic and mutation parameters used in ABC analyses. Notes: Populations i are wild populations (invasive and native) and populations k are biocontrol strains (i.e., laboratory reared populations). Times were translated into numbers of generations running back in time from 2007 by assuming 2.5 generations per year in prior set 1, and 3 generations per year in prior set 2 [19]. NS = stable effective population size (number of diploid individuals); NF = effective number of founders during an introduction step lasting BD generation(s); ar = admixture rate (only for scenarios with admixture); ti = introduction date of invasive populations i with bounds xi or yi fixed from dates of first observation, assuming 2.5 or 3 generations per year, respectively; tbc = creation date of unsampled biocontrol strain for ENA and WNA populations (with condition ti<or = tbci) bounded between the dates of first observation of invasive population (which would correspond to a direct introduction into the wild) and the number of generations from 1970, the start date of a period of intense HA biocontrol activity in the USA. For microsatellite marker parameters, the loci were assumed to follow a generalized stepwise mutation model [S1] with two parameters: the mean mutation rate (mean μ) and the mean parameter of the geometric distribution (mean P) of the length in number of repeats of mutation events. Each locus has a possible range of 40 contiguous allelic states and is characterized by individual μloc and Ploc values, with μloc drawn from a Gamma (mean = mean μ and shape = 2) distribution and Ploc drawn from a Gamma (mean = mean P and shape = 2) distribution [S2]. Uneven insertion/deletion events that were suspected for several of our microsatellite loci based on observed allele sizes (i.e., allele lengths were sometimes not multiple of the motif length implying that there has been insertion-deletion mutations [28]) were also simulated with [file pone.0009743.s005.doc]

**Table S2**

|  | Prior Set 1 |  |  |  |  |  | Prior Set 2 |  |  |  |  |  |
| --- | --- | --- | --- | --- | --- | --- | --- | --- | --- | --- | --- | --- |
| parameters | Distribution | Mean | Median | Mode | Quantile 2.5% | Quantile 97.5% | Distribution | Mean | Median | Mode | Quantile 2.5% | Quantile 97.5% |
| *NS_i_* | Uniform  [100 – 20,000] | 10,056 | 10,040 | NA | 640 | 19,490 | Normal  (10,000 ; 5,000) | 9,993 | 9,990 | 9,980 | 1,640 | 18,340 |
| *NS_k_* | Uniform  [10 – 1,000] | 506 | 508 | NA | 35 | 975 | Normal  (500 ; 250) | 502 | 501 | 498 | 86 | 922 |
| *NF_i_* | Loguniform  [2 – 1,000] | 162 | 45 | 2 | 2 | 862 | Lognormal  (30 ; 30) | 136 | 39 | 44 | 2 | 797 |
| *ar* | Uniform  [0.1 – 0.9] | 0.5 | 0.5 | NA | 0.12 | 0.88 | Normal  (0.5 ; 0.25) | 0.5 | 0.5 | 0.5 | 0.15 | 0.86 |
| *t_i_* | Uniform  [*x_i_* – *x_i_*+5] | DV | DV | NA | DV | DV | Uniform  [*y*_i_ – *y*_i_+5] | DV | DV | NA | DV | DV |
| *tbc_i_* | Loguniform  [*x_i_* – 93] | DV | DV | DV | DV | DV | Loguniform  [*y*_i_ – 111] | DV | DV | DV | DV | DV |
| *BD_i_* | Uniform  [0 – 5] | 2.5 | 2.5 | NA | 0 | 5 | Uniform  [0 – 5] | 2.5 | 2.5 | NA | 0 | 5 |
| mean *µ* | Uniform  [10^-5^ – 10^-3^] | 5.0x10^-4^ | 5.0x10^-4^ | NA | 3.5x10^-5^ | 9.8x10^-4^ | Loguniform  [10^-5^ – 10^-3^] | 2.1x10^-4^ | 1.0x10^-4^ | 1.0x10^-5^ | 1.1x10^-5^ | 8.9x10^-4^ |
| mean *P* | Uniform  [0.1 – 0.3] | 0.2 | 0.2 | NA | 0.10 | 0.29 | Gamma  (30 ; 136) | 0.22 | 0.22 | 0.21 | 0.15 | 0.29 |
| mean *µ*SNI | Uniform  [10^-8^ – 10^-4^] | 5.0x10^-5^ | 5.0x10^-5^ | NA | 2.5x10^-6^ | 9.7x10^-5^ | Loguniform  [10^-8^ – 10^-4^] | 1.1x10^-5^ | 1.0x10^-6^ | 1.0x10^-8^ | 1.3x10^-8^ | 7.9x10^-5^ |
