# Supplementary material for: Bridgehead Effect in the Worldwide Invasion of the Biocontrol Harlequin Ladybird
Source: PLoS One. 2010 Mar 17;5(3):e9743. doi: 10.1371/journal.pone.0009743 (PMC2840033; doi:10.1371/journal.pone.0009743)
Supplement: Table S3 — Posterior probabilities (P) of the selected (most likely) scenarios in each ABC analysis at two different thresholds of smallest Euclidian distances (0.1% and 1%) and two different sets of priors. Notes: Prior sets are detailed in Table S2. 95% confidence intervals (CI) are in brackets. The 95% CI of the selected scenarios never overlapped those of competing scenarios. The values presented in Figure 1 of the main text are those obtained using the 0.1% threshold and prior set 1. (0.05 MB DOC) [file pone.0009743.s006.doc]

**Table S3**

|  | Prior set 1 |  |  | Prior set 2 |  |  |
| --- | --- | --- | --- | --- | --- | --- |
| Invaded area | Selected scenario | P 0.1% | P 1% | Selected scenario | P 0.1% | P 1% |
| East North America (Analysis 1) | Introduction from the native area | 0.999  [0.999 – 1.000] | 0.996  [0.994 – 0.998] | Introduction from the native area | 0.999  [0.999 – 1.000] | 0.999  [0.999 – 1.000] |
| West North America  (Analysis 2) | Introduction from the native area | 0.803  [0.616 – 0.989] | 0.727  [0.653 – 0.800] | Introduction from the native area | 0.953  [0.893 – 1.000] | 0.848  [0.794 – 0.903] |
| Europe  (Analysis 3) | Admixture between eastern North America and European biocontrol | 0.982  [0.921 – 1.000] | 0.951  [0.915 – 0.987] | Admixture between eastern North America and European biocontrol | 0.844  [0.710 – 0.953] | 0.892  [0.813 – 0.972] |
| South America  (Analysis 4) | Introduction from eastern North America | 0.991  [0.980 – 1.000] | 0.979  [0.969 – 0.989] | Introduction from eastern North America | 0.980  [0.944 – 1.000] | 0.977  [0.964 – 0.991] |
| Africa  (Analysis 5) | Introduction from eastern North America | 0.951  [0.819 – 1.000] | 0.844  [0.750 – 0.938] | Introduction from eastern North America | 0.973  [0.849 – 1.000] | 0.754  [0.603 – 0.904] |
