# Supplementary material for: Bridgehead Effect in the Worldwide Invasion of the Biocontrol Harlequin Ladybird
Source: PLoS One. 2010 Mar 17;5(3):e9743. doi: 10.1371/journal.pone.0009743 (PMC2840033; doi:10.1371/journal.pone.0009743)
Supplement: Table S4 — Classical population genetics statistics of the studied HA populations and inferred source populations of the five HA invasive outbreaks. Notes: Mean corrected number of alleles per locus (Na), expected heterozygosity (He) [S3], pairwise F ST matrix and mean individual assignment log-likelihood of invasive populations to putative source populations (in parentheses) [28]. Dashes refer to the pairs of invasive and source populations that are chronologically incompatible. The deduced origin of each outbreak is the sample for which the F ST-value is the smallest and the assignment likelihood is maximized (values in bold). Ebc = European biocontrol strain; ENA = Eastern North America; WNA = Western North America; SA = South America; EU = Europe; AF = Africa. Supporting reference: S3. Nei M (1978) Estimation of average heterozygosity and genetic distance from a small number of individuals. Genetics 89: 583–590. (0.06 MB DOC) [file pone.0009743.s007.doc]

**Table S4**

| Possible source populations |  |  | Invasive outbreaks | | | | |
| --- | --- | --- | --- | --- | --- | --- | --- |
|  | *N_a_* | *H_E_* | ENA | WNA | EU | SA | AF |
| Native area | 5.33 | 0.601 | **0.017 (-18.8)** | **0.011 (-19.9)** | **0.048 (-24.2)** | 0.094 (-24.3) | 0.031 (-20.5) |
| Ebc | 2.94 | 0.431 | 0.188 (-33.2) | 0.184 (-34.4) | 0.111 (-28.9) | 0.279 (-39.4) | 0.188 (-34.1) |
| ENA | 4.55 | 0.553 |  | 0.023 (-22.0) | 0.059 (-27.1) | **0.064 (-18.5)** | **0.023 (-17.6)** |
| WNA | 4.79 | 0.566 | - |  | 0.064 (-27.8) | 0.107 (-23.8) | 0.037 (-21.0) |
| EU | 4.68 | 0.614 | - | - |  | 0.109 (-23.7) | 0.066 (-22.4) |
| SA | 3.47 | 0.496 | - | - | - |  | 0.089 (-23.2) |
| AF | 4.13 | 0.549 | - | - | - | - |  |
| Inferred source based on raw values of *F*_ST_ and assignment statistics |  |  | Native area | Native area | Native area | ENA | ENA |
| Inferred source based on ABC analyses |  |  | Native area | Native area | admixture  EBC + ENA | ENA | ENA |
